# Geometric Phase-Induced Stückelberg Interference in an Optical Lattice Clock

**DOI:** 10.3390/e28080931

**Published:** 2026-08-20

**Authors:** Wei-Xin Liu, Zhan-Peng Lu, Tao Wang

**Affiliations:** 1 Department of Physics, Xinzhou Normal University, Xinzhou 034000, China; 2 Chongqing Key Laboratory for Strongly Coupled Physics, Chongqing University, Chongqing 401331, China; 3 Center of Modern Physics, Institute for Smart City of Chongqing University in Liyang, Liyang 213300, China

**Keywords:** geometric phase, Stückelberg interference, optical lattice clock

## Abstract

We theoretically investigate geometric Stückelberg interferometry in a doubly driven optical lattice clock (OLC). By tuning the relative phase between the two driving fields, we control the relative sign of the effective coupling strengths at the avoided crossings. Within the adiabatic-impulse model, we analyze the time evolution of the two-level system, where nonadiabatic transitions occur only near the crossing points and adiabatic evolution takes place between them. We show that, besides the usual dynamical phase and the Stokes phase, a gauge-invariant noncyclic geometric phase contributes to the final transition probability. This geometric contribution yields a stable π-phase shift in the Stückelberg interference fringes. Moreover, we demonstrate that, under realistic experimental conditions, this geometric Stückelberg interferometer remains insensitive to inhomogeneities in atom-light coupling arising from the finite temperature of the atomic ensemble. Our results provide a general framework for engineering and detecting geometric phases on the OLC platform.

## 1. Introduction

Since its discovery by Berry [1], the geometric phase has attracted substantial interest in both theoretical and experimental studies due to its noise-resilience feature. It has been extensively reviewed in the context of electronic properties [2], and experimentally demonstrated in various platforms, including solid-state spins [3,4] and optical clock transitions [5]. In his original work, Berry pointed out that when a quantum system undergoes a cyclic evolution driven by slowly varying parameters, it acquires, in addition to the familiar dynamical phase, a gauge-invariant phase factor—the geometric phase. This concept was subsequently generalized to noncyclic evolutions, in which the parameters of the Hamiltonian do not return to their initial values [6,7,8]. Compared to the cyclic evolution, such noncyclic behavior may offer distinct technological advantages. For instance, because the system is not required to return to its original state, geometric operations such as geometric quantum gates could potentially be performed faster. This idea was proposed in early theoretical works on noncyclic geometric quantum computation [9,10] and has since been extended through various approaches, including nonadiabatic schemes [11], shortcuts to adiabaticity [12], and generalized gate protocols [13,14]. Experimental realizations have been reported in trapped-ion [15] and superconducting-circuit [16] systems. Additionally, quantum optimal control [17] and quantum metrology [18,19,20] may also stand to benefit.

The noncyclic geometric phase has conventionally been measured with light [21,22,23], neutron [24,25,26], and atom interferometers [27,28]. Recently, Stückelberg interferometry has provided an alternative route to access this phase, notably in topological band structures [29,30,31] and optical lattice systems [32]. Stückelberg interferometry describes the coherent interference that arises when a quantum system is driven repeatedly through an avoided level crossing [33,34,35]. At each avoided crossing, the system—such as a two-level system—undergoes a nonadiabatic process known as the Landau–Zener (LZ) transition [36,37,38]. When the system passes through two avoided crossings in succession, the LZ transitions coherently split and later recombine the wavefunction. Meanwhile the phase accumulated between transitions, which may include the noncyclic geometric phase, manifests as interference in the final transition probability. This approach has been successfully applied to reveal geometric phases from a Stückelberg interferometer formed by two Dirac cones in two-dimensional systems by tuning the relative sign of the masses of the two cones.

**Figure 1:**
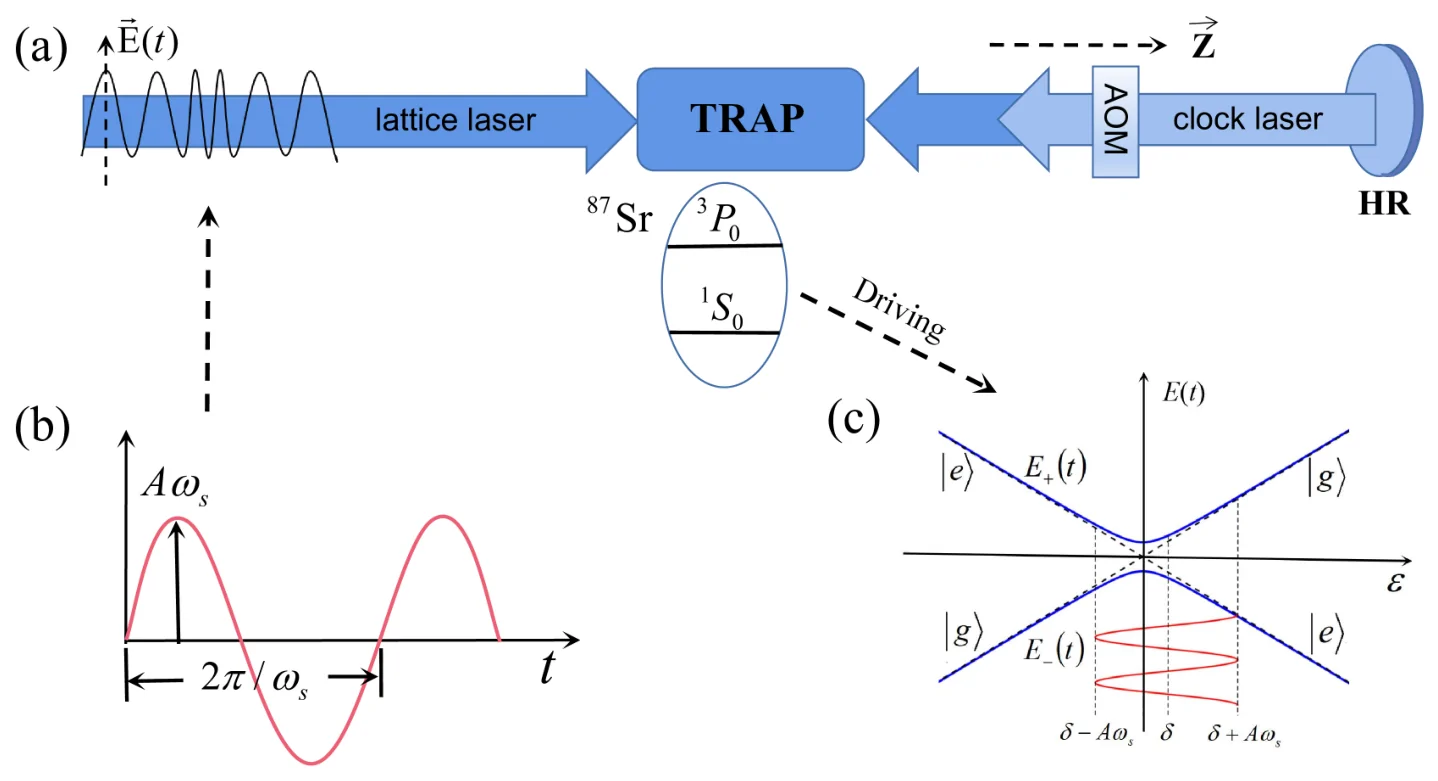
The sketch of the driven optical lattice clock system. (**a**) The one-dimensional optical lattice is constructed with the incident laser laying in the z direction and its reflected laser by the high-reflection mirror (HR), and the clock laser is aligned with the lattice laser to excite the clock transition. An acoustic optical modulator (AOM) is used for simultaneously modulating both the frequency and intensity of the clock laser. (**b**) The frequency modulation of the lattice laser is conducted by adding a sinusoidal driving signal to the piezoelectric transducer. (**c**) Energy levels under the frequency modulation. The two solid blue curves represent the adiabatic energy levels, and the black dashed lines show the crossing diabatic levels. The modulation form is indicated with the red curve.

In this work, we extend this method to an optical lattice clock (OLC). As one of the most precise measurement platforms, the OLC has made considerable progress over the past few years [39,40,41,42,43], and supports a wide range of applications including gravitational redshift tests [44,45], verification of relativistic effects [46,47], and detections of dark matter [48]. The OLC platform is well suited to studying geometric Stückelberg interferometry. Its ultra-narrow clock transition and magic-wavelength trap provide long coherence times and minimize spontaneous-emission losses, which are essential for resolving interference fringes. The lattice and clock laser frequencies can be modulated with high precision, enabling fine control of the relative phase. Meanwhile, the state-dependent coupling allows controlled investigation of thermal averaging effects. Compared with superconducting qubits, trapped ions, or quantum dots, the OLC offers a cleaner and more controllable environment, with well-established techniques for state preparation and detection. These features make it an ideal testbed for geometric-phase physics and its metrological applications. Here, we consider a doubly driven model realized in the ^87^Sr OLC, where both the internal atomic levels and the Rabi frequency are modulated simultaneously (see Figure 1). This scheme has been demonstrated in a recent experiment [49]. By tuning the parameters of the Hamiltonian, the system can pass the avoided crossing points successively (see Figure 1c). In the vicinity of each avoided crossing, we linearize the Hamiltonian, then the nonadiabatic transition can be described by the LZ formula. The relative phase ϕ between the two driving fields allows us to tune the relative sign of the effective coupling strength (this relative sign is determined by the relative phase and plays a key role in the interference pattern. The explicit definitions of the coupling strengths are provided below). With the help of the adiabatic impulse model (AIM) [34,35], we analytically solve the time evolution of the system and get the final transition probability after one time period. We find that, in addition to the well-known dynamical phase (which depends on the eigenenergies) and the Stokes phase acquired at the LZ transitions, a gauge-invariant noncyclic geometric phase also contributes to the final transition probability. As a result, the Stückelberg interference pattern in the transition probability exhibits a geometric π-phase shift relative to the usual case.

We emphasize the difference from previous work [50], in which a cyclic nonadiabatic geometric phase appeared only when the system returned to its initial state after a full period, and asymptotically approached π in the adiabatic limit. Here, we focus on the noncyclic adiabatic geometric phase accumulated between two LZ transitions. This phase does not require the system to return to its initial state and can yield a stable geometric π phase. We further show that, under realistic experimental conditions, this geometric Stückelberg interferometer remains robust against the inhomogeneities in the atom-light coupling that arise from the finite temperature of the atomic ensemble.

The paper is organized as follows. In Section 2, the LZ Hamiltonian model is described. In Section 3, we formulate the Stückelberg interferometry and show that a nontrivial geometric phase appears in the interference pattern. We summarize our main findings and conclude in Section 4.

## 2. Model Hamiltonian

Here, we consider a doubly modulated two-level system in which the relative phase between the two driving modes of the Hamiltonian is tunable. This model was recently realized in a one-dimensional (1D)OLC by simultaneously modulating both the internal levels of the ^87^Sr atom and the Rabi frequency [49]. This 1D optical lattice is constructed with a pair of counter-propagating laser beams, which form a standing-wave potential, laid in z axis (see Figure 1a). After the cold ^87^Sr atoms are loaded into the 1D optical lattice, a modulation is applied to the frequency of the incoming lattice laser by adding a periodic sinusoidal voltage signal (see Figure 1b), and meanwhile the frequency and intensity of the clock laser are simultaneously modulated by use of an acoustic optical modulator (AOM). In addition, the modulations of the lattice frequency and the clock laser frequency are synchronized, ensuring that the relative phase ϕ is stable and finely tunable. Under those modulations, the Hamiltonian of the system takes the form

(1) $$\hat{H}\left(t\right)/\hbar =\frac{\varepsilon (t)}{2}{\hat{\sigma }}_{z}+\frac{\Delta (t)}{2}{\hat{\sigma }}_{x},$$

with $\varepsilon \left(t\right)=\delta +A\omega_{s}\cos\left(\omega_{s}t\right)$ and $\Delta \left(t\right)=g_{\vec{n}}\cos\left(\omega_{s}t+\phi \right)$ are respectively the longitudinal and transverse driving fields, where δ is the frequency difference between the atom internal energy gap and clock laser, $\omega_{s}$ is the driving frequency, *A* is the renormalized dimensionless driving amplitude, $g_{\vec{n}}$ is the atom-light coupling strength of motional states of the optical lattice $|\vec{n}\rangle =|n_{z},n_{x}\rangle$ [49,51,52,53], ϕ is the relative phase between the two driving fields, and ${\hat{\sigma }}_{x,z}$ are the Pauli matrices.

**Figure 2:**
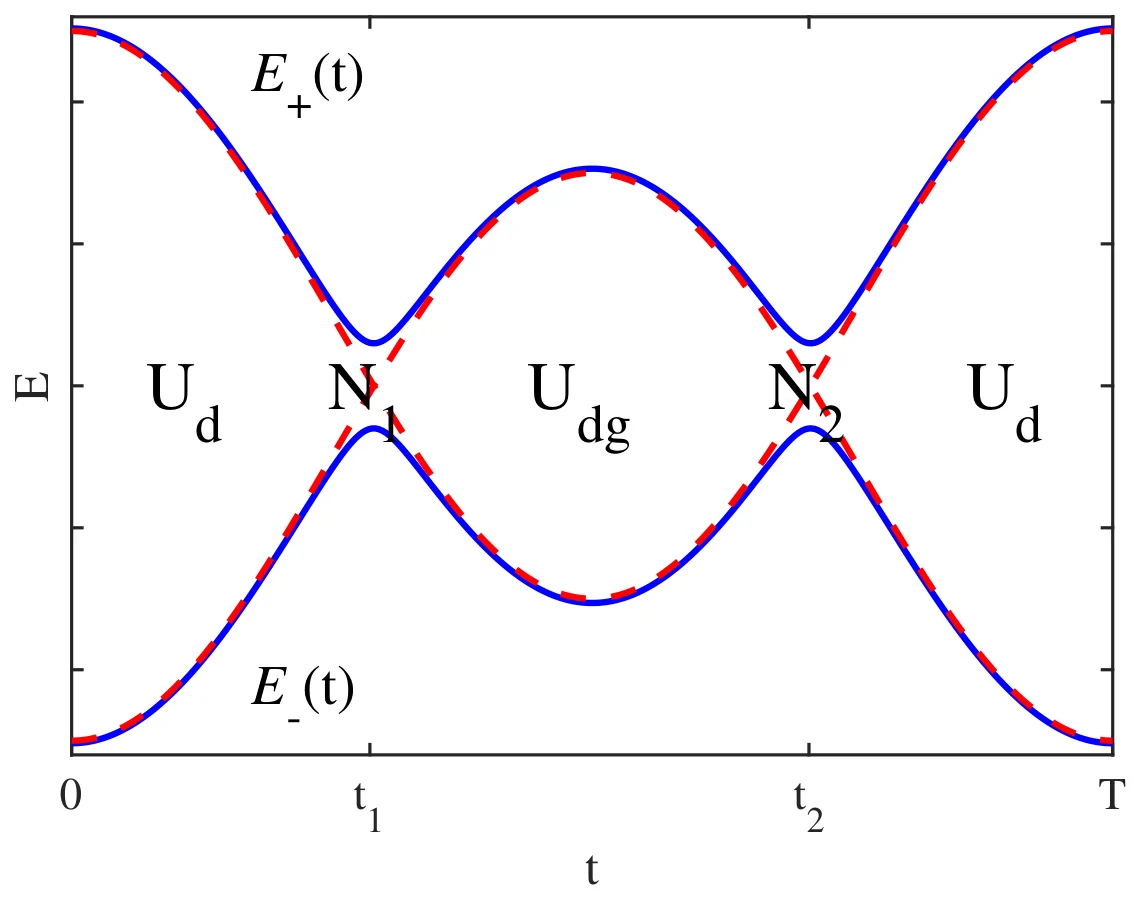
Energy spectrum E(t) as a function of the time *t* over one full period. The solid blue curves denote the adiabatic energy levels $|\varphi_{\pm }\left(t\right)\rangle$, and the dashed red lines indicate the diabatic levels |e〉 and |g〉. The *U*- and *N*-matrices describe the evolution of the system in the corresponding time intervals.

Let |e〉,|g〉 denote the eigenstates of ${\hat{\sigma }}_{z}$ with eigenvalues ±1, which constitute the diabatic basis. Meanwhile the instantaneous eigenstates $|\varphi_{\pm }\left(t\right)\rangle$ of the Hamiltonian Equation (1) satisfy $\hat{H}\left(t\right)|\varphi_{\pm }\left(t\right)\rangle =E_{\pm }\left(t\right)|\varphi_{\pm }\left(t\right)\rangle$, with eigenenergies $E_{\pm }\left(t\right)=\pm \frac{1}{2}\Omega \left(t\right)$, $\Omega \left(t\right)=\hbar \sqrt{\varepsilon^{2}\left(t\right)+\Delta^{2}\left(t\right)}$. $|\varphi_{\pm }\left(t\right)\rangle$ constitute the adiabatic basis and read $|\varphi_{\pm }\left(t\right)\rangle =\beta_{\pm }\left(t\right)|e\rangle \pm \beta_{\mp }\left(t\right)|g\rangle$ with $\beta_{\pm }\left(t\right)=\sqrt{\left[\Omega (t)\pm \varepsilon (t)]/2\Omega (t\right)}$. When $A\omega_{s}>\delta$, the eigenenergies of Hamiltonian Equation (1) exhibit two avoided crossings at $t_{1,2}+nT$, where $\omega_{s}t_{1}=\arccos\left(-\delta /A\omega_{s}\right)$, $\omega_{s}t_{2}=2\pi -\omega_{s}t_{1}$, n∈Z and $T=2\pi /\omega_{s}$, as shown in Figure 2. Note when far away from the avoided crossings, i.e., when |ε(t)|≫|Δ(t)|, the adiabatic eigenstates reduce to the diabatic basis.

In order to study the evolution of the system, we use the so-called AIM, in which one can treat the system as undergoing nonadiabatic transitions only at avoided crossings and as evolving adiabatically between them [54,55]. Provided the nonadiabatic region is sufficiently narrow, we make the approximation by linearizing the Hamiltonian near the avoided crossings, then the nonadiabatic evolution can be obtained by solving the LZ problem. We make a linear expansion around the crossings $t_{1}\left(t_{2}\right)$ and get the linear LZ Hamiltonian

(2) $$\begin{array}{c} {\hat{H}}_{1,2}/\hbar =\mp \frac{vt}{2}{\hat{\sigma }}_{z}+\frac{g_{1,2}}{2}{\hat{\sigma }}_{x}, \end{array}$$

where $v=A\omega_{s}^{2}\sqrt{1-{\left(\delta /A\omega_{s}\right)}^{2}}$, $g_{1}=g_{\vec{n}}\cos\left[\arccos\left(-\delta /A\omega_{s}\right)+\phi \right]$, and $g_{2}=g_{\vec{n}}\cos\left[\arccos\left(-\delta /A\omega_{s}\right)-\phi \right]$ are the effective coupling strengths at the avoided crossings $t_{1}$ and $t_{2}$, respectively. Thus, tuning ϕ from 0 to π/2 changes the sign of $g_{2}$ relative to $g_{1}$. The minus (plus) sign in front of the first term in Equation (2) corresponds to $t_{1(2)}$.

## 3. Stückelberg Interferometry

Now we analyze the time evolution of the system using the AIM. The adiabatic process can be described by standard quantum adiabatic theory, while the instantaneous nonadiabatic transition is approximately governed by the time-independent unitary matrix. To illustrate the geometric phase, here we focus on the two cases ϕ=0 and ϕ=π/2, which correspond to $\mathrm{sgn}\left(g_{1}\right)=\mathrm{sgn}\left(g_{2}\right)$ and $\mathrm{sgn}\left(g_{1}\right)=-\mathrm{sgn}\left(g_{2}\right)$, respectively. For these two cases, the Hamiltonian Equation (2) reduces to the similar form given in Equation (9) of Ref. [32]. Thus the evolution of states at the avoided crossing points $t_{1,2}$ can be described by the unitary evolution *N*-matrix in the adiabatic basis (for simplicity, here we set δ>0, which means $g_{1}<0$) [34,35]

(3) $$\begin{array}{c} N_{1}=\left(\begin{array}{cc} \sqrt{1-P_{\mathrm{LZ}}}e^{-i\varphi_{s}} & \sqrt{P_{\mathrm{LZ}}}\\ -\sqrt{P_{\mathrm{LZ}}} & \sqrt{1-P_{\mathrm{LZ}}}e^{i\varphi_{s}} \end{array}\right) \end{array}$$

and

(4) $$\begin{array}{c} N_{2}=\left(\begin{array}{cc} \mathrm{sgn}(g_{2})\sqrt{1-P_{\mathrm{LZ}}}e^{-i\varphi_{s}} & -\sqrt{P_{\mathrm{LZ}}}\\ \sqrt{P_{\mathrm{LZ}}} & \mathrm{sgn}(g_{2})\sqrt{1-P_{\mathrm{LZ}}}e^{i\varphi_{s}} \end{array}\right), \end{array}$$

where $P_{\mathrm{LZ}}=\exp\left[-2\pi \chi \right]$ is the so-called LZ formula for the non-adiabatic transition, $\chi =g_{1(2)}^{2}/\left(4v\right)$ is the adiabaticity parameter, and $\varphi_{s}=\pi /4+\chi \left(\ln\chi -1\right)+\arg\left[\Gamma \left(1-i\chi \right)\right]$ is the Stokes phase.

Between the two avoided crossings, the system undergoes adiabatic evolution and accumulates a total phase that includes both dynamical and geometric contributions. The adiabatic evolution can be described with the unitary matrix in the adiabatic basis

(5) $$\begin{array}{c} U_{dg}=\left(\begin{array}{cc} e^{-i\left(\frac{\varphi_{d}}{2}-\Gamma_{+}\right)} & 0\\ 0 & e^{i\left(\frac{\varphi_{d}}{2}+\Gamma_{-}\right)} \end{array}\right), \end{array}$$

where $\varphi_{d}=\int_{t_{1}}^{t_{2}}\left[E_{+}\left(t\right)-E_{-}\left(t\right)\right]\;dt$ is the dynamic phase, and

(6) $$\Gamma_{\pm }=\int_{t_{1}}^{t_{2}}\;dt\langle \varphi_{\pm }\left(t\right)|i\partial_{t}|\varphi_{\pm }\left(t\right)\rangle +\arg\langle \varphi_{\pm }\left(t_{1}\right)|\varphi_{\pm }\left(t_{2}\right)\rangle ,$$

where the plus (minus) sign corresponds to the noncyclic geometric phase acquired in the upper (lower) instantaneous eigenstate during the open path from $t_{1}$ to $t_{2}$ [29,30,32]. Thus, as shown in Figure 2, the evolution over a full period is governed by the product of the sequential transfer matrices

(7) $$U_{T}=U_{d}\left(T,t_{2}\right)N_{2}U_{dg}N_{1}U_{d}\left(t_{1},t_{0}\right),$$

where $U_{d}\left(t_{f},t_{i}\right)=\int_{t_{i}}^{t_{f}}\left[E_{+}\left(t\right)-E_{-}\left(t\right)\right]/2\;dt$. Considering the system is prepared in the state |g〉 at $t_{0}=0$—that is, |ψ(0)〉=|g〉—the population probability of the excited state |e〉 after one single period can be described by

(8) $$\begin{array}{cc} P_{e} & =\left|\langle e\right|U_{T}{\left|\psi \left(0\right)\rangle \right|}^{2}\\ \; & =4P_{\mathrm{LZ}}\left(1-P_{\mathrm{LZ}}\right)\sin^{2}\left(\varphi_{s}+\frac{\varphi_{d}}{2}+\frac{\varphi_{g}}{2}\right), \end{array}$$

where $\varphi_{g}=\Gamma_{-}-\Gamma_{+}$ is the geometric phase. We can see the excited state population oscillates as a function of the interference phase $\phi_{\mathrm{Tol}}=\varphi_{s}+\varphi_{d}/2+\varphi_{g}/2$, which includes three parts: a Stokes phase $\varphi_{s}$ acquired during the nonadiabatic transition, a dynamic phase $\varphi_{d}$ and a geometric phase $\varphi_{g}$ acquired adiabatically during the open path $t_{1}$ to $t_{2}$. Note that for the relative phase ϕ=0 and ϕ=π/2, the geometric phase gives two distinct values $\varphi_{g}=0$ and $\varphi_{g}=\pi$, respectively.

## 4. Results in OLC

We now turn to the geometric Stückelberg interference results based on an OLC. A key difference from the single-particle problem is that the cold atoms are trapped in each lattice site and occupy the external motional states according to a Boltzmann distribution. The temperature thus introduces different LZ transition dynamics for atoms in distinct motional states. The transition probability $P_{e}$ in Equation (8) must therefore be thermally averaged over the Boltzmann-weighted population of all external states; that is,

(9) $${\langle P_{e}\rangle }_{T}=\sum_{n_{z},n_{x}}q\left(n_{z}\right)q\left(n_{x}\right)P_{e},$$

where $q\left(n_{z,x}\right)=\frac{e^{-E_{n_{z,x}}/\left(k_{B}T_{z,x}\right)}}{\sum_{n_{z,x}}e^{-E_{n_{z,x}}/\left(k_{B}T_{z,x}\right)}}$ are Boltzmann factors, $E_{n_{z,x}}=h\nu_{z,x}\left(n_{z,x}+1/2\right)$, where $\nu_{z(x)}$ are the longitudinal (transverse) trap frequencies, and $g_{\vec{n}}=ge^{-(\eta_{z}^{2}+\eta_{x}^{2})/2}L_{n_{z}}\left(\eta_{z}^{2}\right)L_{n_{x}}\left(\eta_{x}^{2}\right)$ is the coupling strength in the external state $|n_{z},n_{x}\rangle$, *g* is the bare Rabi frequency, $L_{n}$ is the Laguerre polynomial, and $\eta_{z,x}$ are the Lamb–Dicke parameters. More generally, all the observables $O(\vec{n})$ hereafter in the external state $|\vec{n}\rangle$ should be modified to ${\langle O\rangle }_{T}=\sum_{n_{z},n_{x}}O\left(n_{z},n_{x}\right)q\left(n_{z}\right)q\left(n_{x}\right)$. In order to simulate the experiment, we choose the parameters that are feasible in the 1D ^87^Sr OLC system [49,51,52,53,56], i.e., the longitudinal and transverse frequencies are $\nu_{z}=65$ kHz and $\nu_{x}=250$ Hz, the number of states in the longitudinal and transverse direction are $N_{z}=5$ and $N_{x}=1300$, *T_z_* = *T_x_* = 1 μK, $\eta_{z}≃0.27$, $\eta_{x}≃0.04$.

**Figure 3:**
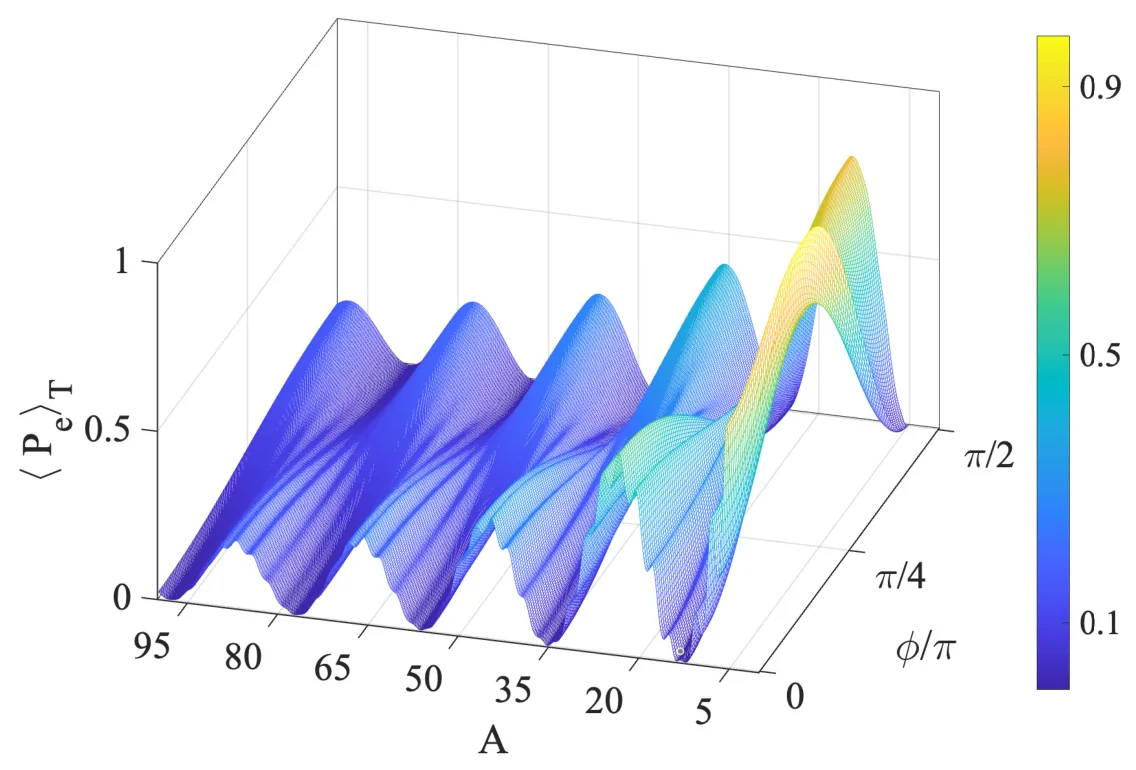
The Stückelberg interference fringes. Numerical excited-state population probability ${\langle P_{e}\rangle }_{T}$ as a function of the driving amplitude *A* and the relative phase ϕ at *T_z_* = *T_x_* = 1 μK.

To highlight the geometric phase shift, we set the parameter $\delta /\omega_{s}=A/\sqrt{2}$. This choice guarantees that $|g_{1}\left|\;=\;\right|g_{2}|$ for both ϕ=0 and ϕ=π/2, and thus makes the dynamical phases as well as the Stokes phases identical in the two cases. The resulting Stückelberg interference fringes are presented in Figure 3. Compared with the Figure 1c of Ref. [29], we plot the transition probability ${\langle P_{e}\rangle }_{T}$ as a function of both the driving amplitude *A* and the relative phase ϕ. The data shown are generated from a numerical solution of Equation (1) based on the Floquet method [57,58] at the temperature *T_z,x_* = 1 μK. We can see that the transition probability ${\langle P_{e}\rangle }_{T}$ shows clear oscillations with *A*, and these oscillations vanish at ϕ=π/4, where $g_{2}=0$. In this case, the Landau–Zener transition probability at the second crossing $t_{2}$ reaches unity ($P_{\mathrm{LZ}}=1$), which completely suppresses the interference contrast. Furthermore, because the sign of $g_{2}$ changes as ϕ varies from 0 to π/2, the interference fringes for ϕ=π/2 exhibit a π phase shift relative to those for ϕ=0.

**Figure 4:**
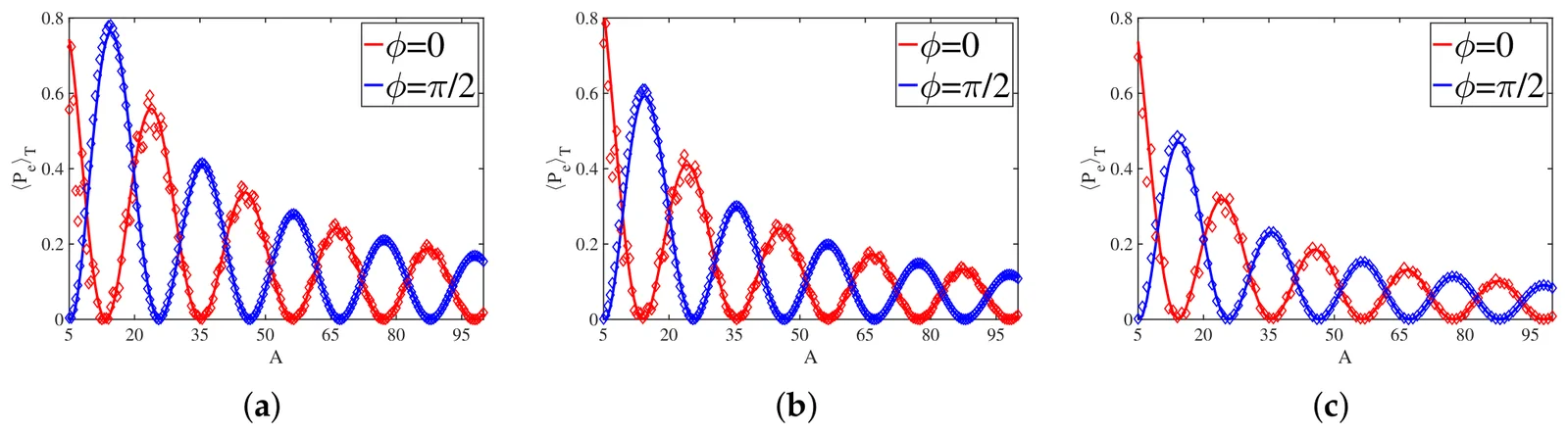
The transition probability ${\langle P_{e}\rangle }_{T}$ as a function of driving amplitude *A* for: (**a**) *T_z,x_* = 1 μK, (**b**) *T_z,x_* = 2 μK, (**c**) *T_z,x_* = 3 μK. The solid lines represent the analytical results obtained from Equations (8) and (9), while the diamond symbols show the numerical Floquet results.

To further investigate the geometric π-shift, we focus on the two special cases ϕ=0 and ϕ=π/2. In these cases, the transition probability $P_{e}$ reduces to $P_{e}\left(\phi =0\right)=4P_{\mathrm{LZ}}\left(1-P_{\mathrm{LZ}}\right)\sin^{2}\left(\varphi_{s}+\frac{\varphi_{d}}{2}\right)$ and $P_{e}\left(\phi =\pi /2\right)=4P_{\mathrm{LZ}}\left(1-P_{\mathrm{LZ}}\right)\cos^{2}\left(\varphi_{s}+\frac{\varphi_{d}}{2}\right)$. Compared with the Figure 7 of Ref. [31] and Figure 2a of Ref. [32], we show the numerical results of the transition probability ${\langle P_{e}\rangle }_{T}$ (marked with the blue and red diamonds) obtained by the Floquet approach, plotted against the amplitude *A* for ϕ=0 and ϕ=π/2 at *T_z,x_* = 1 μK in Figure 4a. We can see that the Stückelberg oscillations exhibit clear $\sin^{2}$ and $\cos^{2}$ behavior, which indicates a distinct geometric π phase shift. The numerical results are in good agreement with the analytical predictions $P_{e}\left(\phi =0\right)$ and $P_{e}\left(\phi =\pi /2\right)$ (marked with the solid red and blue lines). More importantly, this geometric phase shift remains robust against the thermal effect, as verified by increasing the system temperature. In Figure 4b,c, we plot the transition probability ${\langle P_{e}\rangle }_{T}$ as a funtion of *A* at the same conditions except at the temperature *T_z,x_* = 2 μK and *T_z,x_* = 3 μK, respectively. The π-shifted interference fringes remain clearly visible, although the overall transition probability is somewhat reduced.

**Figure 5:**
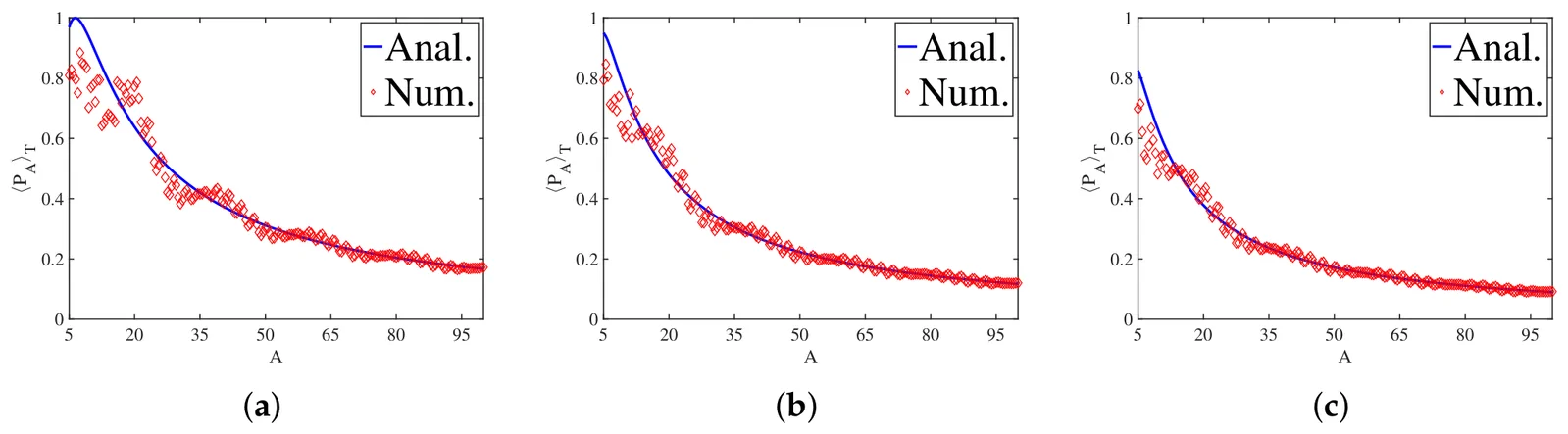
The amplitude of the Stückelberg oscillation ${\langle P_{A}\rangle }_{T}$ as a function of the driving amplitude *A* for: (**a**) *T_z,x_* = 1 μK, (**b**) *T_z,x_* = 2 μK, (**c**) *T_z,x_* = 3 μK. The solid blue line represents the analytical results, while the red diamonds show the numerical results obtained via the Floquet approach.

The amplitude of the Stückelberg oscillations also offers a convenient observable for investigating the π shift, which we consider next. If there is a exact π shift, we would have $P_{A}=P_{e}\left(\phi =0\right)+P_{e}\left(\phi =\pi /2\right)\equiv 4P_{\mathrm{LZ}}\left(1-P_{\mathrm{LZ}}\right)$ (note that here we have ensured the dynamic phases and the Stokes phases are same for ϕ=0 and ϕ=π/2), which is independent of the phase factors. To verify this relation, we show in Figure 5a the amplitude of oscillation ${\langle P_{e}\left(\phi =0\right)+P_{e}\left(\phi =\pi /2\right)\rangle }_{T}$ as a function of *A* with the numerical Floquet approach (presented in the red diamonds), in analogy with Figure 2b of Ref. [32]. We can see that the numerical data follow the analytical curve ${\langle P_{A}\rangle }_{T}$ (presented by the solid blue line) closely, confirming the predicted π shift. We also plot ${\langle P_{A}\rangle }_{T}$ at *T_z,x_* = 2 μK and *T_z,x_* = 3 μK in Figure 5b,c. These results also demonstrate the robustness of the geometric π shift against thermal effects.

From an experimental perspective, the predicted π-phase shift can be detected by measuring the transition probability $P_{e}$ as a function of the relative phase ϕ for the two cases ϕ=0 and ϕ=π/2. The $\sin^{2}$ and $\cos^{2}$ forms of the interference pattern directly reveal the π shift between them. Since the dynamical and Stokes phases are kept identical for both cases, the observed shift can be unambiguously attributed to the geometric contribution. To distinguish this geometric shift from other possible phase offsets—such as laser phase noise, relative phase drift between the two driving fields, systematic calibration errors, or detection-phase offsets—we suggest the following strategies. First, a differential measurement comparing the two cases cancels common phase contributions. Second, the case ϕ=π/4, where $g_{2}=0$ and the interference contrast is suppressed, provides a useful reference point for calibrating and removing experimental offsets. Third, the oscillation amplitude ${\langle P_{A}\rangle }_{T}$ provides an additional temperature-insensitive signature, as it eliminates the oscillatory phase factors entirely and reduces to a constant independent of ϕ.

## 5. Conclusions and Discussion

We have presented a theoretical analysis of geometric Stückelberg interferometry in a doubly driven optical lattice clock. Within the adiabatic-impulse model, we derived the transition probability for a two-level system traversing two consecutive avoided crossings and identified the emergence of a gauge-invariant noncyclic geometric phase as a distinct contribution alongside the dynamical phase and the Stokes phase. Unlike the cyclic geometric phase reported in earlier work, which requires the system to return to its initial state and approaches π only in the adiabatic limit, the phase we consider here is noncyclic in nature, accumulated along an open path between two LZ transitions, and can yield a stable geometric π shift under appropriate parameter conditions. A central result of our analysis is that this geometric π shift manifests clearly in the Stückelberg interference fringes. By tuning the relative phase ϕ between the two driving fields, we effectively reverse the sign of the effective coupling at the second avoided crossing, which in turn switches the interference from a form. This behavior is explicitly captured in our analytical expressions and confirmed by the numerical Floquet approach. The close agreement between the analytical and numerical results validates our theoretical treatment and confirms that the geometric phase contribution is not merely a theoretical artifact but a physically observable quantity.

An important practical aspect of our work concerns the thermal effects inherent in the OLC platform. The atoms are distributed over the external motional states according to a Boltzmann distribution, and each motional state experiences slightly different LZ transition dynamics due to the state-dependent coupling strength. This inhomogeneity might, in principle, wash out the interference pattern entirely, as different motional states contribute oscillations with different amplitudes and phases. Our thermal averaging procedure shows that, while the overall transition probability is reduced at higher temperatures, the π-shifted interference pattern persists. The underlying reason is that the geometric phase is independent of the motional quantum number–it depends only on the relative sign of the couplings, not on their magnitude. This topological robustness against thermal broadening suggests that the geometric Stückelberg interferometer remains operational under realistic experimental conditions without requiring ground-state cooling or single-site resolution. Moreover, the oscillation amplitude provides a direct and temperature-insensitive signature of the geometric phase, as it eliminates the oscillatory phase factors entirely. In addition, our noncyclic scheme does not rely on the system returning to its initial state, which not only simplifies the experimental requirements but also offers greater flexibility for potential applications in quantum control. In this context, our scheme can be viewed as a continuous-time analogue of the nonadiabatic noncyclic geometric gates that have been proposed for superconducting qubits and trapped ions [59,60]. The difference lies in the fact that here the geometric phase is accumulated naturally in the course of periodic driving, rather than being synthesized through a sequence of pulsed operations. The ability to engineer a stable geometric phase without completing a closed loop in parameter space may prove advantageous for geometric quantum gates, where speed and resilience to control errors are critical.

Our findings point to several avenues worth exploring in future studies. First, while we have focused on the ^87^Sr OLC as a concrete implementation, the same theoretical framework should be applicable to other driven two-level systems, such as superconducting qubits or semiconductor quantum dots, provided the avoided crossings are well separated in time. In fact, the condition for the adiabatic-impulse model to hold—namely, that the nonadiabatic regions are narrow compared to the separation between crossings—is satisfied in a broad class of driven systems, suggesting that the geometric phase effect we describe may be universal. Second, the interplay between the geometric phase and decoherence mechanisms beyond thermal dephasing—such as collisional shifts or laser noise—deserves more detailed treatment. The robustness we have demonstrated against thermal effects does not guarantee insensitivity to other types of noise, particularly those that couple with the relative phase ϕ between the two driving fields. A systematic study of the interferometer’s response to different noise spectra would be valuable for assessing its practical viability. Third, it would be interesting to explore whether the noncyclic geometric phase can be exploited for quantum sensing, where its topological nature might offer protection against certain types of noise. Because the geometric phase is gauge-invariant and independent of dynamical details, it could serve as a robust signal for detecting external fields or perturbations that affect the relative phase between the two driving modes. In this sense, the OLC platform—already a leading system for precision metrology—could simultaneously serve as a detector of geometric-phase shifts, opening a new avenue for hybrid quantum sensing that combines frequency metrology with topological protection [18]. Fourth, one could extend the analysis to multi-level systems or to systems with multiple avoided crossings, where the interference pattern may exhibit richer geometric contributions, possibly including non-Abelian geometric phases. Such extensions would connect our work to the broader field of geometric and topological quantum computation, where the noncommutativity of geometric phases offers a route to fault-tolerant quantum information processing. Additionally, it is worth noting that fractional calculus has recently emerged as a powerful tool for describing anomalous transport and nonlocal effects in low-dimensional materials [61]. Although the direct application of such methods to driven two-level systems remains unexplored, the fractional modeling framework may offer a promising avenue for capturing non-Markovian or memory effects in quantum interferometric setups—a direction we leave for future investigation.

We now summarize the main advantages and limitations of our method. On the positive side, the scheme is operationally simple, requiring only synchronized modulations and phase tuning that are already feasible in current OLCs. The geometric π shift is robust against thermal effects, as the geometric phase is independent of motional quantum numbers. The noncyclic nature eliminates the need to return to the initial state, offering flexibility and potential speed advantages for gate operations. The AIM also applies broadly to other driven two-level systems [34,35]. On the other hand, the two-level approximation may not capture all features of real multi-level systems. Other decoherence sources—such as laser phase noise or intensity fluctuation—require further investigation. While feasible in the laboratory, implementation in transportable systems may be more challenging due to stricter phase stability requirements. Finally, the stable π shift is obtained only under specific parameter conditions, and generalization to broader regimes remains open. These limitations naturally point to future directions, as discussed above.

In summary, our work establishes a clear theoretical foundation for observing and manipulating noncyclic geometric phases via Stückelberg interferometry in optical lattice clocks. More broadly, our results illustrate how a seemingly minor modification—from cyclic to noncyclic evolution—can lead to qualitatively distinct physical behavior and open up new possibilities for both fundamental studies and practical applications. The robustness of the π shift against thermal averaging, combined with the analytical tractability of the model, positions the OLC as a versatile testbed for geometric-phase physics and its applications in precision measurement and quantum information.

## Data Availability

The original contributions presented in this study are included in the article. Further inquiries can be directed to the corresponding author.

## References

[1] Berry M.V. (1984). Quantal phase factors accompanying adiabatic changes. Proc. R. Soc. Lond. A.

[2] Xiao D., Chang M.-C., Niu Q. (2010). Berry phase effects on electronic properties. Rev. Mod. Phys..

[3] Zu C., Wang W.-B., He L., Zhang W.-G., Dai C.-Y., Wang F., Duan L.-M. (2014). Experimental realization of universal geometric quantum gates with solid-state spins. Nature.

[4] Huang Y.-Y., Wu Y.-K., Wang F., Hou P.-Y., Wang W.-B., Zhang W.-G., Lian W.-Q., Liu Y.-Q., Wang H.-Y., Zhang H.-Y. (2019). Experimental Realization of Robust Geometric Quantum Gates with Solid-State Spins. Phys. Rev. Lett..

[5] Zaporski L., Liu Q., Velez G., Radzihovsky M., Li Z., Colombo S., Pedrozo-Penafiel E., Vuletić V. (2025). Quantum-amplified global-phase spectroscopy on an optical clock transition. Nature.

[6] Samuel J., Bhandari R. (1988). General Setting for Berry’s Phase. Phys. Rev. Lett..

[7] Weinfurter H., Badurek G. (1990). Measurement of Berry’s phase for noncyclic evolution. Phys. Rev. Lett..

[8] Wang X.-B., Kwek L.C., Liu Y., Oh C.H. (2001). Noncyclic phase for neutrino oscillation. Phys. Rev. D.

[9] Friedenauer A., Sjöqvist E. (2003). Noncyclic geometric quantum computation. Phys. Rev. A.

[10] Wang Z.S., Liu G.Q., Ji Y.H. (2009). Noncyclic geometric quantum computation in a nuclear-magnetic-resonance system. Phys. Rev. A.

[11] Liu B.-J., Su S.-L., Yung M.-H. (2020). Nonadiabatic noncyclic geometric quantum computation in Rydberg atoms. Phys. Rev. Res..

[12] Lv Q.-X., Liang Z.-T., Liu H.-Z., Liang J.-H., Liao K.-Y., Du Y.-X. (2020). Noncyclic geometric quantum computation with shortcut to adiabaticity. Phys. Rev. A.

[13] Eivarsson N., Sjöqvist E. (2023). Genuinely noncyclic geometric gates in two-pulse schemes. Phys. Rev. A.

[14] Qin Z.-H., Yuan Y.-H., Liang Y., Xue Z.-Y., Chen T. (2026). Robustness enhancement of universal noncyclic geometric gates via evolution optimization. Phys. Rev. A.

[15] Zhang J.W., Yan L.-L., Li J.C., Ding G.Y., Bu J.T., Chen L., Su S.-L., Zhou F., Feng M. (2021). Single-Atom Verification of the Noise-Resilient and Fast Characteristics of Universal Nonadiabatic Noncyclic Geometric Quantum Gates. Phys. Rev. Lett..

[16] Qiu L., Li H., Han Z., Zheng W., Yang X., Dong Y., Song S., Lan D., Tan X., Yu Y. (2021). Experimental realization of noncyclic geometric gates with shortcut to adiabaticity in a superconducting circuit. Appl. Phys. Lett..

[17] Chen T., Xue Z.-Y. (2020). High-Fidelity and Robust Geometric Quantum Gates that Outperform Dynamical Ones. Phys. Rev. Appl..

[18] Zhou Z., Carrasco S.C., Sanner C., Malinovsky V.S., Folman R. (2025). Geometric phase amplification in a clock interferometer for enhanced metrology. Sci. Adv..

[19] Aharonov Y., Kaufherr T., Popescu S., Reznik B. (1998). Quantum Measurement Backreaction and Induced Topological Phases. Phys. Rev. Lett..

[20] Cho Y.-W., Kim Y., Choi Y.-H., Kim Y.-S., Han S.-W., Lee S.-Y., Moon S., Kim Y.-H. (2019). Emergence of the geometric phase from quantum measurement back-action. Nat. Phys..

[21] Morinaga A., Monma A., Honda K., Kitano M. (2007). Berry’s phase for a noncyclic rotation of light in a helically wound optical fiber. Phys. Rev. A.

[22] Leinonen A., Hannonen A., Partanen H., Heikkinen J., Setälä T., Friberg A.T., Hakala T.K. (2023). Noncyclic continuous Pancharatnam—Berry phase in dual-beam interference. Commun. Phys..

[23] van Dijk T., Schouten H.F., Ubachs W., Visser T.D. (2010). The Pancharatnam-Berry phase for non-cyclic polarization changes. Opt. Express.

[24] Filipp S., Hasegawa Y., Loidl R., Rauch H. (2005). Noncyclic geometric phase due to spatial evolution in a neutron interferometer. Phys. Rev. A.

[25] Wagh A.G., Rakhecha V.C., Fischer P., Ioffe A. (1998). Neutron Interferometric Observation of Noncyclic Phase. Phys. Rev. Lett..

[26] Klepp J., Sponar S., Hasegawa Y., Jericha E., Badurek G. (2005). Noncyclic Pancharatnam phase for mixed state SU(2) evolution in neutron polarimetry. Phys. Lett. A.

[27] Morinaga A., Nanri K. (2012). Noncyclic Berry phase and scalar Aharonov-Bohm phase for the spin-redirection evolution in an atom interferometer. Phys. Rev. A.

[28] Zhou Z., Margalit Y., Moukouri S., Meir Y., Folman R. (2020). An experimental test of the geodesic rule proposition for the noncyclic geometric phase. Sci. Adv..

[29] Lim L.-K., Fuchs J.-N., Montambaux G. (2014). Mass and Chirality Inversion of a Dirac Cone Pair in Stückelberg Interferometry. Phys. Rev. Lett..

[30] Lim L.-K., Fuchs J.-N., Montambaux G. (2015). Geometry of Bloch states probed by Stückelberg interferometry. Phys. Rev. A.

[31] Lim L.-K., Fuchs J.-N., Montambaux G. (2015). Geometric phase in Stückelberg interferometry. Phys. Rev. A.

[32] Shen X., Li Z. (2018). Topological characterization of a one-dimensional optical lattice with a force. Phys. Rev. A.

[33] Stückelberg E.C.G. (1932). Theory of Inelastic Collisions between Atoms Theory of Inelastic Collisions between Atoms, Using Two Simultaneous Differential Equations. Helv. Phys. Acta.

[34] Shevchenko S.N., Ashhab S., Nori F. (2010). Landau-Zener-Stückelberg interferometry. Phys. Rep..

[35] Ivakhnenko O.V., Shevchenko S.N., Nori F. (2023). Nonadiabatic Landau-Zener-Stückelberg-Majorana transitions, dynamics, and interference. Phys. Rep..

[36] Landau L.D. (1932). Zur Theorie der Energieubertragung II. Phys. Z. Sowjetunion.

[37] Zener C. (1932). Non-adiabatic crossing of energy levels. Proc. R. Soc. A.

[38] Majorana E. (1932). Atomi orientati in campo magnetico variabile. Nuovo Cimento.

[39] Ludlow A.D., Boyd M.M., Ye J., Peik E., Schmidt P.O. (2015). Optical atomic clocks. Rev. Mod. Phys..

[40] Aeppli A., Kim K., Warfield W., Safronova M.S., Ye J. (2024). Clock with 8 × 10^−19^ Systematic Uncertainty. Phys. Rev. Lett..

[41] Liu X.-Y., Liu P., Li J., Zhang Y.-C., Wang Y.-B., Jia Z.-P., Zhang X., Zhu X.-Q., Kong D.-Q., Song W.-L. (2025). Zero-Dead-Time Strontium Lattice Clock with a Stability at 10^−19^ Level. Phys. Rev. Lett..

[42] Fortier T.M., Luiten A.N., Margolis H.S. (2026). Optical Atomic Clocks: Defining the Future of Time and Frequency Metrology. Optica.

[43] Bothwell T., Hunt B.D., Siegel J.L., Hassan Y.S., Grogan T., Kobayashi T., Gibble K., Porsev S.G., Safronova M.S., Brown R.C. (2025). Lattice Light Shift Evaluations in a Dual-Ensemble Yb Optical Lattice Clock. Phys. Rev. Lett..

[44] Bothwell T., Kennedy C.J., Aeppli A., Kedar D., Robinson J.M., Oelker E., Staron A., Ye J. (2022). Resolving the gravitational redshift across a millimetre-scale atomic sample. Nature.

[45] Zheng X., Dolde J., Cambria M.C., Lim H.M., Kolkowitz S. (2023). A lab-based test of the gravitational redshift with a miniature clock network. Nat. Commun..

[46] Delva P., Lodewyck J., Bilicki S., Bookjans E., Vallet G., Le Targat R., Pottie P.-E., Guerlin C., Meynadier F., Le Poncin-Lafitte C. (2017). Test of Special Relativity Using a Fiber Network of Optical Clocks. Phys. Rev. Lett..

[47] Takamoto M., Ushijima I., Ohmae N., Yahagi T., Kokado K., Shinkai H., Katori H. (2020). Test of General Relativity by a Pair of Transportable Optical Lattice Clocks. Nat. Photonics.

[48] Filzinger M., Dörscher S., Lange R., Klose J., Steinel M., Benkler E., Peik E., Lisdat C., Huntemann N. (2023). Improved Limits on the Coupling of Ultralight Bosonic Dark Matter to Photons from Optical Atomic Clock Comparisons. Phys. Rev. Lett..

[49] Lu X.-T., Wang T., Li T., Zhou C.-H., Yin M.-J., Wang Y.-B., Zhang X.-F., Chang H. (2021). Doubly Modulated Optical Lattice Clock: Interference and Topology. Phys. Rev. Lett..

[50] Liu W.-X., Wang T., Li W.-D. (2023). Nonadiabatic geometric phase in a doubly driven two-level system. Chin. Phys. B.

[51] Blatt S., Thomsen J.W., Campbell G.K., Ludlow A.D., Swallows M.D., Martin M.J., Boyd M.M., Ye J. (2009). Rabi spectroscopy and excitation inhomogeneity in a one-dimensional optical lattice clock. Phys. Rev. A.

[52] Yin M.-J., Wang T., Lu X.-T., Li T., Wang Y.-B., Zhang X.-F., Li W.-D., Smerzi A., Chang H. (2021). Rabi Spectroscopy and Sensitivity of a Floquet Engineered Optical Lattice Clock. Chin. Phys. Lett..

[53] Liu W.-X., Wang T., Zhang X.-F., Li W.-D. (2021). Time-domain Landau-Zener-Stückelberg-Majorana interference in an optical lattice clock. Phys. Rev. A.

[54] Damski B., Zurek W.H. (2006). Adiabatic-impulse approximation for avoided level crossings: From phase-transition dynamics to Landau-Zener evolutions and back again. Phys. Rev. A.

[55] Tomka M., Campos Venuti L., Zanardi P. (2018). Accuracy of the adiabatic-impulse approximation for closed and open quantum systems. Phys. Rev. A.

[56] Tan W., Liu W.-X., Chen Y.-X., Zhou C.-H., Zhao G.-D., Chang H., Dai J., Wang T. (2025). Realization of Landau-Zener Rabi oscillations on an optical lattice clock. Phys. Rev. A.

[57] Shirley J.H. (1965). Solution of the Schrödinger Equation with a Hamiltonian Periodic in Time. Phys. Rev..

[58] Eckardt A. (2017). Colloquium: Atomic quantum gases in periodically driven optical lattices. Rev. Mod. Phys..

[59] Zhu J., Kwek L.C., Xue Z.-Y., Shen L.T., Chen Y.-H., Yang Z.B., Zhou F., Feng M. (2023). Geometric and Holonomic Quantum Computation. Phys. Rep..

[60] Xue Z.-Y., Ding C.-Y. (2026). Recent Advances on Nonadiabatic Geometric Quantum Computation. Front. Phys..

[61] Hernández-Acosta M.A., Martines-Arano H., Soto-Ruvalcaba L., Martínez-González C.L., Martínez-Gutiérrez H., Torres-Torres C. (2020). Fractional Thermal Transport and Twisted Light Induced by an Optical Two-Wave Mixing in Single-Wall Carbon Nanotubes. Int. J. Therm. Sci..

